# Network analysis of depressive and anxiety symptoms in adolescents during the later stage of the COVID-19 pandemic

**DOI:** 10.1038/s41398-022-01838-9

**Published:** 2022-03-10

**Authors:** Hong Cai, Wei Bai, Huanzhong Liu, Xu Chen, Han Qi, Rui Liu, Teris Cheung, Zhaohui Su, Jingxia Lin, Yi-lang Tang, Todd Jackson, Qinge Zhang, Yu-Tao Xiang

**Affiliations:** 1 Unit of Psychiatry, Department of Public Health and Medicinal Administration, & Institute of Translational Medicine, Faculty of Health Sciences, University of Macau, Macao SAR, China; 2 Centre for Cognitive and Brain Sciences, University of Macau, Macao SAR, China; 3 Institute of Advanced Studies in Humanities and Social Sciences, University of Macau, Macao SAR, China; 4 Department of Psychiatry, Chaohu Hospital of Anhui Medical University, Hefei, Anhui Province China; 5 The National Clinical Research Center for Mental Disorders & Beijing Key Laboratory of Mental Disorders, Beijing Anding Hospital & the Advanced Innovation Center for Human Brain Protection, Capital Medical University, Beijing, China; 6 School of Nursing, Hong Kong Polytechnic University, Hong Kong SAR, China; 7 Center on Smart and Connected Health Technologies, Mays Cancer Center, School of Nursing, UT Health San Antonio, San Antonio, TX USA; 8 Department of Rehabilitation Sciences, Hong Kong Polytechnic University, Hong Kong SAR, China; 9 Department of Psychiatry and Behavioral Sciences, Emory University, Atlanta, GA USA; 10 Atlanta Veterans Affairs Medical Center, Decatur, GA USA; 11 Department of Psychology, Faculty of Social Sciences, University of Macau, Macao SAR, China

**Keywords:** Depression, Scientific community

## Abstract

Network analysis is an effective approach for examining complex relationships between psychiatric symptoms. This study was designed to examine item-level relationships between depressive and anxiety symptoms using network analysis in an adolescent sample and identified the most central symptoms within the depressive-anxiety symptoms network model. Depressive and anxiety symptoms were assessed using the Patient Health Questionire-9 (PHQ-9) and Generalized Anxiety Disorder Screener (GAD-7), respectively. The structure of depressive and anxiety symptoms was characterized using “Strength” and “Bridge Strength” as centrality indices in the symptom network. Network stability was tested using a case-dropping bootstrap procedure. Finally, a Network Comparison Test (NCT) was conducted to examine whether network characteristics differed on the basis of gender, school grade and residence. Network analysis revealed that nodes PHQ2 (“Sad mood”), GAD6 (“Irritability”), GAD3 (“Worry too much”), and PHQ6 (“Guilty”) were central symptoms in the network model of adolescents. Additionally, bridge symptoms linking anxiety and depressive symptoms in this sample were nodes PHQ6 (“Guilty”), PHQ2 (“Sad mood”), and PHQ9 (“Suicide ideation”). Gender, school grade and residence did not significantly affect the network structure. Central symptoms (e.g., Sad mood, Irritability, Worry too much, and Guilty) and key bridge symptoms (e.g., Guilty, Sad mood, and Suicide ideation) in the depressive and anxiety symptoms network may be useful as potential targets for intervention among adolescents who are at risk for or suffer from depressive and anxiety symptoms.

## Introduction

Adolescence is a crucial period in life, characterized by many unique changes and challenges. One in six people fall within this age group (i.e., 10–19 years) and half of all psychiatric conditions start before 14 years of age [1]. It is estimated that one in seven adolescents experience mental health problems [2], of which anxiety and depression are the most common disturbances. For example, the estimated 1-year prevalence of depression among adolescents is 4–5% [3–5], while the corresponding figure for anxiety ranges between 5% and 10% [6]. Depression and anxiety often occur together in adolescents [7]. For instance, due to lockdown and school closures during the COVID-19 pandemic in early 2020 [8–10], prevalence estimates of depression and anxiety were 57.0% and 36.7%, respectively, among adolescents in China [10]. Depression and anxiety account for 16% of the global burden of disease for adolescents worldwide [1, 11, 12] and are among the major contributing factors for disability, substance use, self-harm, and suicide behaviors among adolescents [13–16].

Comorbid depression and anxiety have been widely examined with traditional conceptualizations of psychopathology that rely on total scale scores to describe symptom severity. Unfortunately, such approaches may obscure meaningful associations between individual symptoms [17]. Network analysis has emerged as a novel approach to conceptualizing psychological phenomena in a manner that addresses limitations of the traditional approach. In network theory, central symptoms are more likely to activate other symptoms and play a major role in causing the onset and/or maintenance of a syndrome/disorder. Network analysis has the potential to map specific relationships among individual symptoms of a disorder and identify targets for treatment [18]. Furthermore, network analysis can be used to extract the structure of psychiatric disturbances from clinical data [19, 20] and highlight meaningful associations between individual symptoms within and/or between disorders [21]. Additionally, network model is useful in understanding the mechanism of comorbidities and provide hints for clinicians to prevent and treat comorbidities [22].

Network analysis has been used to understand symptom-symptom relationships in psychiatric comorbidities. For example, a network analysis of the Sequenced Treatment Alternatives to Relieve Depression (STAR*D) study revealed the importance of “Sad mood” and “Anhedonia” in non-psychotic depressive disorder [23]. In another network analysis study, “Sad mood” and “Worry” emerged as the most central symptoms in the depression-anxiety network among psychiatric patients [24, 25]. A network analysis on depressed US adolescents (*N* = 1409) revealed that “Self-hatred”, “Loneliness”, “Sadness”, and “Pessimism” were the most central symptoms [26]. Another network analysis on depressive and anxiety symptoms of adolescents in Sub-Saharan Africa found that the most central symptoms were “Guilty” and “Sad mood” in the depressive symptom community, while “Too much worry”, “Uncontrollable worry”, and “Nervousness” were the central symptoms in the anxiety symptom community [27].

Clinical features of depression and anxiety are closely associated with sociocultural and economic factors [28]. Therefore, findings based on samples from Western countries and Africa are not necessarily as applicable within sociocultural and economic contexts of highly populated, rapidly developing Asian countries such as China [29, 30]. To date, no network analysis studies have been published on comorbid depressive and anxiety symptoms in general samples of adolescents in China. Hence, this study examined the item-level relationships between depressive and anxiety symptoms using network analysis in a sample of Chinese adolescents.

## Methods

### Participants and procedure

#### Study design

This cross-sectional study was conducted between 8 August 2020 and 12 March 2021, using snowball sampling method through the collaborative research network of the National Clinical Research Center for Mental Disorders, China. Eligible participants were (1) secondary school students residing in China during the COVID-19 pandemic, who were (2) able to understand the purpose and contents of the assessment. To avoid contagion during the COVID-19 pandemic, following previous studies [31, 32] data were collected online using the WeChat-based “Questionnaire Star” program. WeChat is a widely used smartphone-based social communication APP, with more than 1.2 billion active users in China. All participants (and their caregivers for participants younger than 18 years) provided electronic written informed consent prior to participation in this study. This study was approved by the Institutional Review Board (IRB) of Beijing Anding Hospital.

#### Measures

Severity of the depressive symptoms was assessed using the Chinese version of the 9-item Patient Health Questionnaire (PHQ-9) [33, 34] each item reflected a symptom of depression and was rated from “0” (not at all) to “3” (nearly every day). Total PHQ-9 scores ranged from 0 to 27, with higher scores indicating more severe depressive symptoms. Severity of anxiety symptoms was measured using the Chinese version of the 7-item Generalized Anxiety Disorder Scale (GAD-7) [35, 36]. Each GAD-7, item described a common anxiety symptom and was scored from 0 (not at all) to 3 (nearly every day); total scores ranged from 0 to 21 with higher scores indicating more severe anxiety symptoms.

### Statistical analysis

#### Network analysis

All analyses were conducted using R (Version 4.0.3) [37]. Means, standard deviations (SDs), kurtosis, and skewness of all PHQ-9 and GAD-7 item scores were inspected. Following previous studies [26, 38], the informativeness of each item was estimated by the mean of the standard deviation, and then possible item redundancy was evaluated using “networktools” R package [39]. Due to controversies over the optimal method of modeling scale item scores in network analysis [40], following previous studies [20] the values of all PHQ-9 and GAD-7 items were dichotomized as “0” or “1”, representing the absence or presence of depressive and anxiety symptoms, respectively. Item values of 1, 2, or 3 were converted to “presence” of depressive and anxiety symptoms, respectively, while values of 0 reflected an “absence” of symptoms. An Ising model was used to assess the depressive-anxiety symptoms network structure based on binary data [20, 41]. Briefly, an Ising model can be conceived as a series of pairwise associations between binary variables, after controlling for the confounding effects of all other associations.

To estimate and visualize the network, R-package “qgraph” (Version 1.6.5) [42] and “bootnet” (Version 1.4.3) [43] were used. The network structure was estimated using the Enhanced Least Absolute Shrinkage and Selection Operator (eLASSO) method, which combines a logistic regression analysis with an optimization process to determine the best connection method for each symptom. To identify relationships between nodes, the eLASSO combined logistic regression with model selection based on a Goodness-of-Fit measure. This algorithm could result in a sparse network model which is more interpretable than the original model. Model selection was based on the Extend Bayesian Information Criterion (EBIC) [44, 45]. The binary network was fitted using the R-package “IsingFit” 0.3.1 [20]. When each node (representing a symptom) is connected to a range of other nodes through edges with different weights, the final network is constructed [46]. The thickness of an edge represents the strength of an association. The color of an edge indicates the direction of the association (e.g., green edges indicate positive associations; red edges indicate negative associations). The network is visualized using the Fruchterman-Reingold algorithm [43]. Nodes with stronger and more frequent associations with another node are placed closer with each other and are more concentrated in the network. Network analysis can provide quantitative centrality indicators for each node based on the unique configuration of the network. The predictability of each node was estimated using the R package “mgm”. Predictability was defined as the variance in a node that is explained by all other nodes in the network.

Following previous studies [43, 47], the centrality index of strength was used to indicate importance of individual symptoms in the model; certain other centrality indices, such as betweenness and closeness, are unsuitable as measure of node importance in psychological networks [48] and were excluded. Strength is the sum of the correlations of one node to all other nodes with higher values reflecting greater centrality in the network. Centrality measures are reported as standardized values (“*z* scores”).

#### Estimation of network accuracy and stability

According to recommendations of Epskamp et al. [43], the robustness of the network solution was assessed by estimating the accuracy of edge weights and the stability of centrality indices with the R-package “bootnet” (Version 1.4.3) [43]. The accuracy of edge weights was estimated by computing confidence intervals (CIs) with a non-parametric bootstrapping method [49]. Next, observations in the dataset were resampled randomly to create new datasets from which the 95% CIs were calculated. Larger CIs suggested reduced precision in the estimation of edges while narrower CIs indicated a more trustworthy network [43]. When “0” was included within the range of constructed CIs, this indicated the edge weights (or node strength) of two different symptoms did not significantly differ from each other. In this network analysis, we performed 1000 permutations and used a bootstrap differential test to evaluate differences in network properties, which were used to determine differences between edge weights and between node centrality indexes [26].

Correlation stability coefficients (CS-C) assessed the stability of centrality indexes (i.e., Strengths) using subset bootstraps [50]. If the strength of nodes did not change significantly after excluding a subset of the sample in the dataset, the network structure was considered to be stable. CS-C values represented the maximum proportion of samples that could be removed, while there was a 95% probability that the correlation between original centrality indices could reach at least 0.70 [43]. Generally, the CS-C should not be <0.25, and preferably above 0.50. Subsequently, the difference between two strength indices was considered significant if 1000-bootstrap 95% non-parametric CIs did not contain “0”. Bootstrapped difference tests were used to evaluate differences in network properties [43]. This test relied on 95% CIs to determine whether or not two edge weights or the strength of two nodes significantly differed from one-another. The R package “bootnet” was used to perform the analyses [51].

#### Comparison of network characteristics

Considering the moderating effects of gender, school grade, and residence on anxiety and depressive symptoms among adolescents [52], depression-anxiety network models were compared between genders, between school grades (junior/senior secondary school) and between residences (urban/rural areas). For these analyses, we used Network Comparison Tests (NCT), which are permutation tests that assess the difference between two networks, using the R-package “NetworkComparisonTest” 2.0.1 [53]. NCTs were performed on subsamples (i.e., females vs. males, junior secondary school vs. senior secondary school, and urban vs. rural areas) with 1000 permutations to assess global network strengths (absolute sums of all edge weights) and network structures (distributions of edge weights) between the two networks. In addition, the strength of each edge between the two networks was assessed using Holm-Bonferroni correlations for multiple comparisons.

## Results

### Study sample

Altogether, 1183 adolescents were invited to participate in this study; of these 1057 met study inclusion criteria and completed the assessment. A majority was female (60.3%) and their mean age was 16.30 years (SD = 3.61 years). The sample mean PHQ-9 and GAD-7 total scores were 3.98 (SD = 5.44) and 2.67 (SD = 4.40), respectively (Table 1). Means, SDs, skewness and kurtosis of all PHQ-9 and GAD-7 item scores are presented in Supplementary Table 1. Distribution of the answer to each PHQ-9 and GAD-7 question are presented in Supplementary Table 2. A correlation matrix of PHQ-9 and GAD-7 item scores is presented in Supplementary Table 3.

**Table 1:** Demographic characteristics of the study sample (*n* = 1057).

Variables	
Age, mean (SD)	16.30 (3.61)
Female gender, *n* (%)	637 (60.3%)
School grade, *n* (%)	
Junior secondary school	479 (45.3%)
Senior secondary school	578 (54.7%)
PHQ-9 total, mean (SD)	3.98 (5.44)
GAD total, mean (SD)	2.67 (4.40)

*PHQ-9* nine-item Patient Health Questionnaire, *GAD-7* seven-item Generalized Anxiety Disorder scale.

### Network structure and centrality measures analysis

Tests of item informativeness and redundancy indicated no item ratings were <2.5 SD from the mean level for informativeness (*M*_SD_ = 0.44 ± 0.04); as such, none of the items were poor vis a vis informativeness. Moreover, no items were redundant with any other items (<25% of statistically different correlations). Therefore, all PHQ-9 and GAD-7 items were retained in the analyses.

**Fig. 1:**
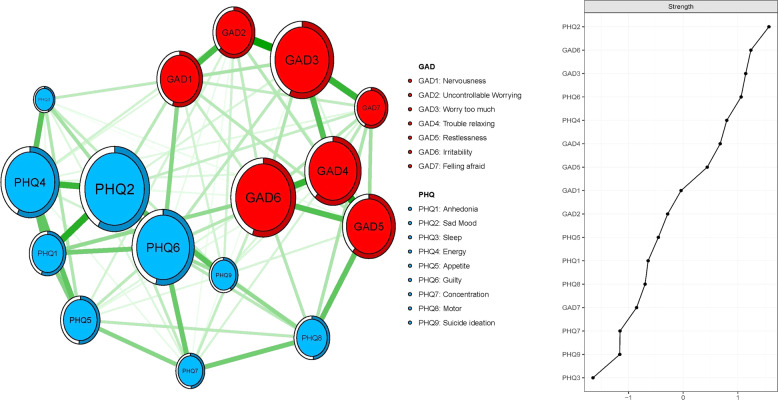
Estimated network model for depressive and anxiety symptoms in adolescents. Ring-shaped pie charts represent predictability (a fully filled dark ring would indicate that 100% of the symptom’s variance is explained by its intercorrelations with the other symptoms in the network). In the diagram symptom node with stronger connections are closer to each other. The blue node denotes the PHQ-9 items (9 -items Patients Health Questionnaire); the red node denotes the GAD-7 items (7-items Generalized Anxiety Disorder scale). The dark green lines represent positive correlations. The edge thickness represents the strength of the association between symptom nodes.

Figure 1 shows the network analysis of depressive and anxiety symptoms using the Ising model. The predictability of symptoms is shown in the form of ring-shaped pie charts in Fig. 1. Mean predictability was 0.54 in this adolescent sample. Within the depressive symptom community, node PHQ1 (“Anhedonia”) had the most direct connection with the node PHQ2 (“Sad mood”), followed by the connection between nodes PHQ2 (“Sad mood”) and PHQ4 (“Energy”), and the connection between nodes PHQ3 (“Sleep”) and PHQ4 (“Energy”).

Within the anxiety symptom community, the node GAD3 (“Worry too much”) had the most direct connection with node GAD2 (“Uncontrollable Worrying”), followed by the connection between nodes GAD4 (“Trouble relaxing”) and GAD5 (“Restlessness”), the connection between nodes GAD3 (“Worry too much”) and GAD7 (“Feeling afraid”) and the connection between nodes GAD4 (“Trouble relaxing”) and GAD6 (“Irritability”). In the depressive and anxiety symptoms network model of Chinese adolescents, node GAD5 (“Restlessness”) was most strongly associated with node PHQ8 (“Motor”) (average edge Weight=1.09), followed by connections between nodes GAD1 (“Nervousness”) and PHQ6 (“Guilty”) (average edge weight = 0.84), and nodes PHQ1 (“Anhedonia”) and GAD6 (“Irritability”) (average edge Weight=0.79) (Fig. 1 and Supplementary Table S3).

In terms of strength, node PHQ2 (“Sad mood”) had the highest strength. Nodes GAD6 (“Irritability”), GAD3 (“Worry too much”), and PHQ6 (“Guilty”) were also statistically stronger than most other symptoms in the network (Fig. 1). Therefore, these four symptoms were central symptoms for understanding the association between depressive and anxiety symptoms in this sample. In contrast, several other symptoms were marginal including nodes PHQ7 (“Concentration”), PHQ9 (“Suicide ideation”) and PHQ3 (“Sleep”) (Fig. 1).

**Fig. 2:**
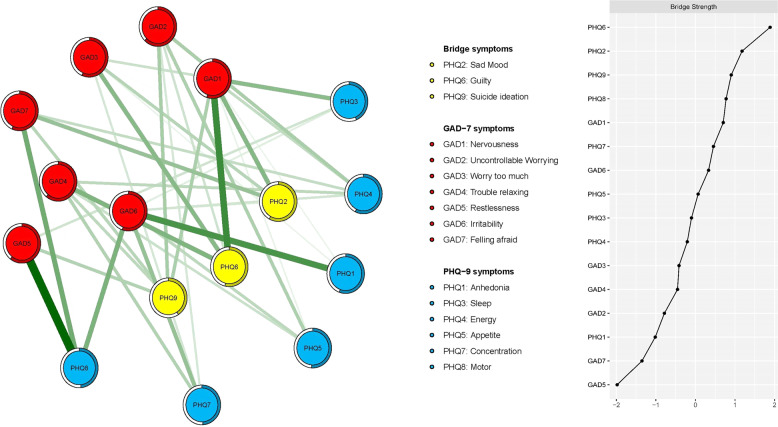
Network structure of depressive and anxiety in adolescents only showing bridge connection. Ring-shaped pie charts represent predictability (a fully filled dark ring would indicate that 100% of the symptom’s variance is explained by its intercorrelations with the other symptoms in the network). In the diagram symptom node with stronger connections are closer to each other. The blue node denotes the PHQ-9 items (9-items Patients Health Questionnaire); the red node denotes the GAD-7 items (7-items Generalized Anxiety Disorder scale). The dark green lines represent positive correlations. The edge thickness represents the strength of the association between symptom nodes.

Following previous studies [22, 54], bridge strength, the best index for identifying nodes in which deactivation could prevent activation spread from one disorder to another was used to identify bridge symptoms. Nodes PHQ6 (“Guilty”), PHQ2 (“Sad mood”) and PHQ9 (“Suicide ideation”) emerged as the three most prominent bridge symptoms (Fig. 2).

**Fig. 3:**
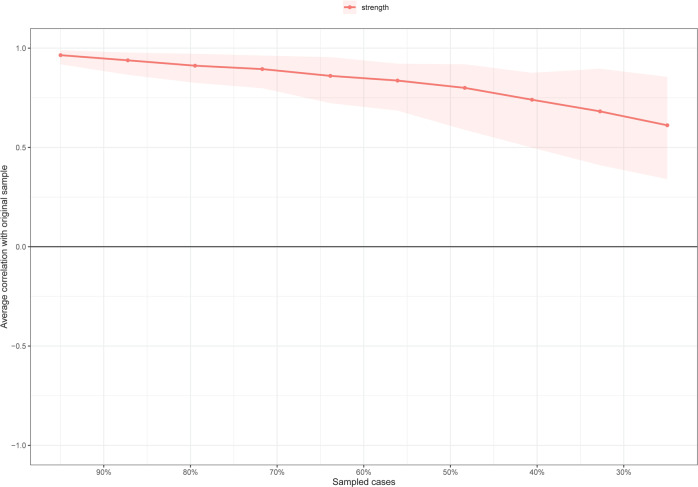
Stability of centrality indices by case dropping subset bootstrap. The x-axis represents the percentage of cases of the original sample used at each step. The y-axis represents the average of correlations between the centrality indices in the original network and the centrality indices from the re-estimated networks after excluding increasing percentages of cases. The line indicates the correlations of strength and bridge strength.

For stability of the network analysis, strength had an excellent level of stability (i.e., CS-coefficient = 0.517), indicating that 51% of the sample could be dropped without significant changes in the network structure (Fig. 3). Bootstrapped 95% CIs for estimated edge-weights suggested that the estimates were reliable and stable (Fig. S1). The bootstrap difference test showed that most of the comparisons between edge weights are statistically significant.

### Network Comparison Tests

In the comparison of network models between female and male adolescents, there were no significant differences in network global strength (network strength among male participants: 7.52 versus female participants: 7.60, *S* = 0.08, *p* = 0.484), but there was a significant difference in edge weights (*M* = 0.28, *p* = 0.022; Supplementary Figs. S2–S4). Three edge weights, PHQ4 (“Energy”) – PHQ8 (“Motor”), PHQ9 (“Suicide ideation”) – GAD1 (“Nervousness”), and PHQ1(“Anhedonia”) - GAD5 (“Restlessness”) were stronger in female than in male adolescents. Conversely, two edge weights, PHQ1 (”Anhedonia”) – PHQ4 (“Energy”) and PHQ8 (“Motor”) - GAD5 (“Restlessness”) were stronger in male than in female adolescents. Subdividing the sample according to junior versus senior secondary school grade, no significant differences were found in network global strength (network strength among junior participants: 7.47; among senior participants: 7.40; *S* = 0.07, *p* = 0.527) or the distribution of edge weights (*M* = 0.18, *p* = 0.626, Supplementary Figs. S5–S7). Subdividing the sample according to urban versus rural residence, no significant differences were found in network global strength (network strength among urban participants: 7.36; among rural participants: 7.61; *S* = 0.25, *p* = 0.108) or the distribution of edge weights (*M* = 0.23, *p* = 0.222, Supplementary Figs. S8–S10).

## Discussion

To our knowledge, this is the first network analysis study of depressive and anxiety symptoms in a general sample of adolescents in China. Analyses indicated nodes PHQ2 (“Sad mood”), GAD6 (“Irritability”), GAD3 (“Worry too much”) and PHQ6 (“Guilty”) were central symptoms in the network model. Additionally, bridge symptoms linking anxiety and depressive symptoms in this sample were nodes PHQ6 (“Guilty”), PHQ2 (“Sad mood”) and PHQ9 (“Suicide ideation”). Gender, school grade and residence did not significantly affect the overall network structure.

“Sad mood” (PHQ2) was one of the most central symptoms to emerge within the depression-anxiety network of Chinese adolescents. Similar findings have been reported in previous studies on depressive and anxiety symptom networks of adults with psychiatric disorders [24, 55], adults with depression [23, 56], a sample of US children and adolescents [25], and adolescents in Sub-Saharan Africa [27]. Our findings also supported sad mood as a required, core symptom for the diagnosis of major depressive disorder (MDD) in line with the Diagnostic and Statistical Manual of Mental Disorder-5 (DSM-5); [57] and International Classification of Diseases, Tenth Revision (ICD-10) [58]. Other studies have also reported the presence of sad mood contributes to the prediction of MDD and increases risk for MDD onset or recurrence [23, 59, 60]. Furthermore, a substantial proportion of Chinese adolescents are “left-behind children” (e.g., children who are left at home for at least half a year while one or both parents move elsewhere to work) who often suffered from financial, social-emotional, behavioral, and educational difficulties [61, 62], which could increase the likelihood of sad mood in adolescents.

The symptom, “worry too much” (GAD3), was another prominent central symptom in the depressive-anxiety symptom network of Chinese adolescents as indicated by its strength. This finding is also consistent with previous findings of depressive and anxiety symptoms network in an adult psychiatric sample [24, 55] and adolescents in Sub-Saharan Africa [27]. “Worry too much” is a hallmark symptom required for a generalized anxiety disorder diagnosis in the DSM-5 [57] and ICD-10 [58]. In this sample, sources of worry could include concerns of academic performance during the COVID-19 pandemic [8, 63–66], infection risk for oneself, one’s family, classmates, and friends [67, 68], and further COVID-19 outbreaks [63]. Together, stressors associated with COVID-19 may increase uncertainty and ambiguity in negotiating tasks of adolescences such as the completion of academic requirements and transitions to further education or work and contribute to chronic worry [69, 70], particularly among already distressed cohorts.

“Irritability” (GAD3) was another prominent central symptom in this network analysis, and is also a key criterion, both for depression in children and adolescents and anxiety disorders in the DSM-5 [57]. In addition, irritability was found to be a predictor of future depression and anxiety disorders in a 20-year follow-up study of a community-based sample [71]. Irritability in adolescents has been linked to increased activity in the insula, prefrontal cortex, and inferior parietal lobule [72]. Abnormalities in both reward and threat processing underlie the clinical presentation of irritability, which includes a greater propensity toward affective (e.g., frustration and anger) and behavioral (e.g., motor activity and aggression) responses [73].

Guilt or negative self-referential thinking has also been implicated in the onset and/or maintenance of depression among adolescents [74, 75]. The emergence of Guilty (PHQ6) as a central symptom in the network of our adolescent sample parallels findings in depressed adolescents in Sub-Saharan Africa [27] and North America [26] and suggests regret about having done or not done something one believes should be done is common among depressed-anxious adolescents across different cultures. Guilt and/or negative self-concept are key features in the DSM-5 diagnosis of major depression [57]. Negative self-evaluations reflecting regrets may increase rumination and attentional focus toward negative self-information [76]. Therefore, negative self-evaluations may contribute to the development and/or maintenance of a depressive episode with co-occurring anxiety [76].

Comorbid depressive and anxiety symptoms are often associated with poor treatment efficacy, and increased rates of hospitalization and disability [77]. We found that “Guilty” (PHQ6), “Sad mood” (PHQ2) and “Suicide ideation” (PHQ9) were key bridge symptoms in the current depression-anxiety network. Similar findings have been found in previous studies wherein “guilty” and “sad mood” have had a role linking the depression and anxiety of between ages 5 and 14 years old [25]. These bridge symptoms do not necessarily extend to older groups, however, as another study found that psychomotor agitation, concentration problems, and restlessness were the bridge symptoms in depression and anxiety network of adults [56]. The discrepancy between studies could be partly due to using different measures of depression and anxiety, though an intriguing hypothesis for future work is the possibility that different clinical features are critical to the co-occurrence of depression within samples of adolescents versus adults. Some studies also found that uncaring family environments, parental rejection, and over-control, unhealthy living style, peer victimization and hopelessness are associated with higher risk of sad mood, suicidal ideation and guilt in adolescents [78–82].

Previous meta-analysis on depressive and anxiety symptoms in children and adolescents found that female sex was associated with increased risk of depressive and anxiety symptoms during COVID-19 pandemic [52]. However, network analysis revealed that only certain edges were different between genders during the late stage of the COVID-19 pandemic, which may be partly due to gender differences involving biological susceptibility, self-esteem, experience of interpersonal violence, and exposure to stress [83].

Identifying central symptoms and bridge symptoms within the depressive-anxiety symptom network model has possible clinical significance; targeting these symptoms may contribute to prevention among at-risk adolescents and improve the effectiveness of treatments targeting co-occurring depressive and anxiety symptom [56]. For example, cognitive behavioral therapies (CBTs) targeting central symptoms and bridge symptoms including “Guilty”, “Excessive worry”, “Sad mood”, and “Irritability” via strategies such as cognitive restructuring and distribution may rapidly improve depressive and anxiety symptoms among adolescents and reduce risk of comorbidity [84, 85]. Furthermore, a recent meta-analysis of 17 randomized control trials (RCTs) concluded that antidepressants are more effective than CBT in treating specific symptoms of depression including “depressed mood”, “feelings of guilt”, “suicidal thoughts”, and “psychic anxiety” [86], though this difference was limited to those patients with the highest elevations on these symptoms and it was not clear how applicable these results were to adolescents given the mean age of just under 40 years across the set of studies. Nonetheless, such findings underscore the need for further research on antidepressants as a potentially viable alternative for targeting key symptoms of depressive and anxiety among adolescents.

Possible implications aside, the main limitations of this study should be noted. First, findings may not be generalized to adolescents in clinical settings, as previous studies have suggested differences in network connectivity may exist in different populations or samples [87]. Furthermore, although some parallels were observed between the network characteristics of the current sample and of adolescents in other countries, generalizations across cultures and contexts (e.g., to a post-COVID-19 world) are tentative at best. Second, due to the cross-sectional research design, dynamic changes and causality between individual symptoms could not be explored. The “snapshot” of the depressive-anxiety symptom network of adolescents provided in this research provides a foundation for more costly, time-consuming longitudinal extensions in future. Third, because symptom assessments were based on self-reports, biases in recall and/or social desirability cannot be ruled out as influences on the results. Fourth, data collected by snowball sampling were used to construct a network model, which could limit the sample representativeness. Fifth, for logistical reasons, data prior to and in the early stage of the COVID-19 pandemic were not collected; therefore, comparisons of symptom patterns in adolescents between different stages of the pandemic could not be conducted.

In conclusion, this study is the first to identify central symptoms (e.g., Sad mood, Irritability, Worry too much, Guilty) and key bridge symptoms (e.g., Guilty, Sad mood, Suicide ideation) within the depression-anxiety network of Chinese adolescents. Findings may provide an impetus for future studies examining symptom networks in other groups of adolescents as a means of clarifying key symptoms that extend across adolescents from different cultures and those that are unique to particular groups. In addition, central symptoms and bridge symptoms identified in this study may be useful targets for interventions designed to prevent these disturbances among at-risk Chinese adolescents and treat those who are currently suffering from co-occurring depressive and anxiety symptoms.

## Supplementary information

supplementary material
