# Supplementary material for: Network analysis of depressive and anxiety symptoms in adolescents during the later stage of the COVID-19 pandemic
Source: Transl Psychiatry. 2022 Mar 10;12:98. doi: 10.1038/s41398-022-01838-9 (PMC8907388; doi:10.1038/s41398-022-01838-9)
Supplement: Supplementary file 1 — supplementary material [file 41398_2022_1838_MOESM1_ESM.docx]

**Supplementary Material**

Table S1. Means, standard deviations, skewness, and kurtosis

Table S2. The distributions of the responses to PHQ-9 and GAD-7 items

Table S3. Correlation matrix of the PHQ-9 and GAD-7 items

Figure S1. Nonparametric bootstrapped difference test

Figure S2. Estimated network model between males and females

Figure S3. Comparison of network centrality indices between females and males.

Figure S4. Comparison of network properties between females and males.

Figure S5. Estimated network model between junior and senior students.

Figure S6. Comparison of network centrality indices between junior and senior students.

Figure S7. Comparison of network properties between junior and senior students.

Figure S8. Estimated network model between urban and rural students.

Figure S9. Comparison of network centrality indices between urban and rural students.

Figure S10. Comparison of network properties between urban and rural students.

Table S1. Means, standard deviations, skewness, and kurtosis of the PHQ-9 and GAD-7 item scores

| Item | M | SD | Skewness | kurtosis |
| --- | --- | --- | --- | --- |
| PHQ1 | 0.38 | 0.49 | 0.48 | -1.77 |
| PHQ2 | 0.37 | 0.48 | 0.53 | -1.72 |
| PHQ3 | 0.31 | 0.46 | 0.81 | -1.35 |
| PHQ4 | 0.37 | 0.48 | 0.53 | -1.73 |
| PHQ5 | 0.27 | 0.45 | 1.02 | -0.96 |
| PHQ6 | 0.34 | 0.47 | 0.66 | -1.56 |
| PHQ7 | 0.29 | 0.45 | 0.93 | -1.13 |
| PHQ8 | 0.20 | 0.40 | 1.53 | 0.34 |
| PHQ9 | 0.12 | 0.32 | 2.36 | 3.58 |
| GAD1 | 0.27 | 0.44 | 1.04 | -0.91 |
| GAD2 | 0.23 | 0.42 | 1.26 | -0.42 |
| GAD3 | 0.31 | 0.46 | 0.80 | -1.36 |
| GAD4 | 0.28 | 0.45 | 0.99 | -1.01 |
| GAD5 | 0.20 | 0.40 | 1.52 | 0.30 |
| GAD6 | 0.30 | 0.46 | 0.86 | -1.26 |
| GAD7 | 0.20 | 0.40 | 1.51 | 0.28 |

Table S2. The distributions of the responses to PHQ-9 and GAD-7 items

| Item | Answers n (%) | |
| --- | --- | --- |
|  | Absence | Presence |
| PHQ-9 |  |  |
| PHQ1 | 652 (61.7) | 405 (38.3) |
| PHQ2 | 665 (62.9) | 392 (37.1) |
| PHQ3 | 727 (68.8) | 330 (31.2) |
| PHQ4 | 663 (62.7) | 394 (37.3) |
| PHQ5 | 769 (72.8) | 288 (27.2) |
| PHQ6 | 695 (65.8) | 362 (34.2) |
| PHQ7 | 752 (71.1) | 305 (28.9) |
| PHQ8 | 850 (80.4) | 207 (19.6) |
| PHQ9 | 932 (88.2) | 125 (11.8) |
| GAD-7 |  |  |
| GAD1 | 773 (73.1) | 284 (26.9) |
| GAD2 | 810 (76.6) | 247 (23.4) |
| GAD3 | 725 (68.6) | 332 (31.4) |
| GAD4 | 764 (72.3) | 293 (27.7) |
| GAD5 | 848 (80.2) | 209 (19.8) |
| GAD6 | 738 (69.8) | 319 (30.2) |
| GAD7 | 847 (80.1) | 210 (19.9) |

Note: GAD, Generalized Anxiety Disorder; PHQ, Patient Health Questionnaire.

Table S3. Correlation matrix of the PHQ-9 and GAD-7 item scores.

|  | PHQ-1 | PHQ-2 | PHQ-3 | PHQ-4 | PHQ-5 | PHQ-6 | PHQ-7 | PHQ-8 | PHQ-9 | GAD-1 | GAD-2 | GAD-3 | GAD-4 | GAD-5 | GAD-6 | GAD-7 |
| --- | --- | --- | --- | --- | --- | --- | --- | --- | --- | --- | --- | --- | --- | --- | --- | --- |
| PHQ1 | 0 |  |  |  |  |  |  |  |  |  |  |  |  |  |  |  |
| PHQ2 | 1.47 | 0 |  |  |  |  |  |  |  |  |  |  |  |  |  |  |
| PHQ3 | 0.24 | 0.67 | 0 |  |  |  |  |  |  |  |  |  |  |  |  |  |
| PHQ4 | 1.09 | 1.34 | 1.14 | 0 |  |  |  |  |  |  |  |  |  |  |  |  |
| PHQ5 | 0.87 | 0 | 0.59 | 1.09 | 0 |  |  |  |  |  |  |  |  |  |  |  |
| PHQ6 | 0.98 | 0.91 | 0 | 0.40 | 0.52 | 0 |  |  |  |  |  |  |  |  |  |  |
| PHQ7 | 0 | 0.50 | 0.32 | 0.25 | 0.85 | 1.03 | 0 |  |  |  |  |  |  |  |  |  |
| PHQ8 | 0 | 0.19 | 0.12 | 0.13 | 0.54 | 0.54 | 0.98 | 0 |  |  |  |  |  |  |  |  |
| PHQ9 | 0 | 1.13 | 0.48 | 0 | 0.30 | 0.70 | 0 | 0.78 | 0 |  |  |  |  |  |  |  |
| GAD1 | 0.12 | 0.54 | 0.52 | 0.29 | 0.14 | 0.84 | 0 | 0 | 0.38 | 0 |  |  |  |  |  |  |
| GAD2 | 0 | 0 | 0 | 0.39 | 0.38 | 0.24 | 0.21 | 0 | 0.40 | 1.25 | 0 |  |  |  |  |  |
| GAD3 | 0.13 | 0.34 | 0.26 | 0.05 | 0 | 0.53 | 0.22 | 0 | 0 | 0.63 | 1.70 | 0 |  |  |  |  |
| GAD4 | 0 | 0 | 0 | 0.31 | 0.26 | 0.56 | 0.36 | 0.05 | 0.32 | 0 | 0.27 | 1.19 | 0 |  |  |  |
| GAD5 | 0 | 0 | 0.24 | 0 | 0 | 0 | 0 | 1.09 | 0.36 | 0.55 | 0.56 | 0.33 | 1.67 | 0 |  |  |
| GAD6 | 0.79 | 0.17 | 0.17 | 0.23 | 0.32 | 0 | 0.47 | 0.58 | 0 | 0.49 | 0.50 | 0.59 | 1.37 | 1.19 | 0 |  |
| GAD7 | 0 | 0.47 | 0 | 0.29 | 0 | 0 | 0 | 0.62 | 0.34 | 0.50 | 0.11 | 1.37 | 0.54 | 0.70 | 0.56 | 0 |

Figure S1. Nonparametric bootstrapped difference test.

A:


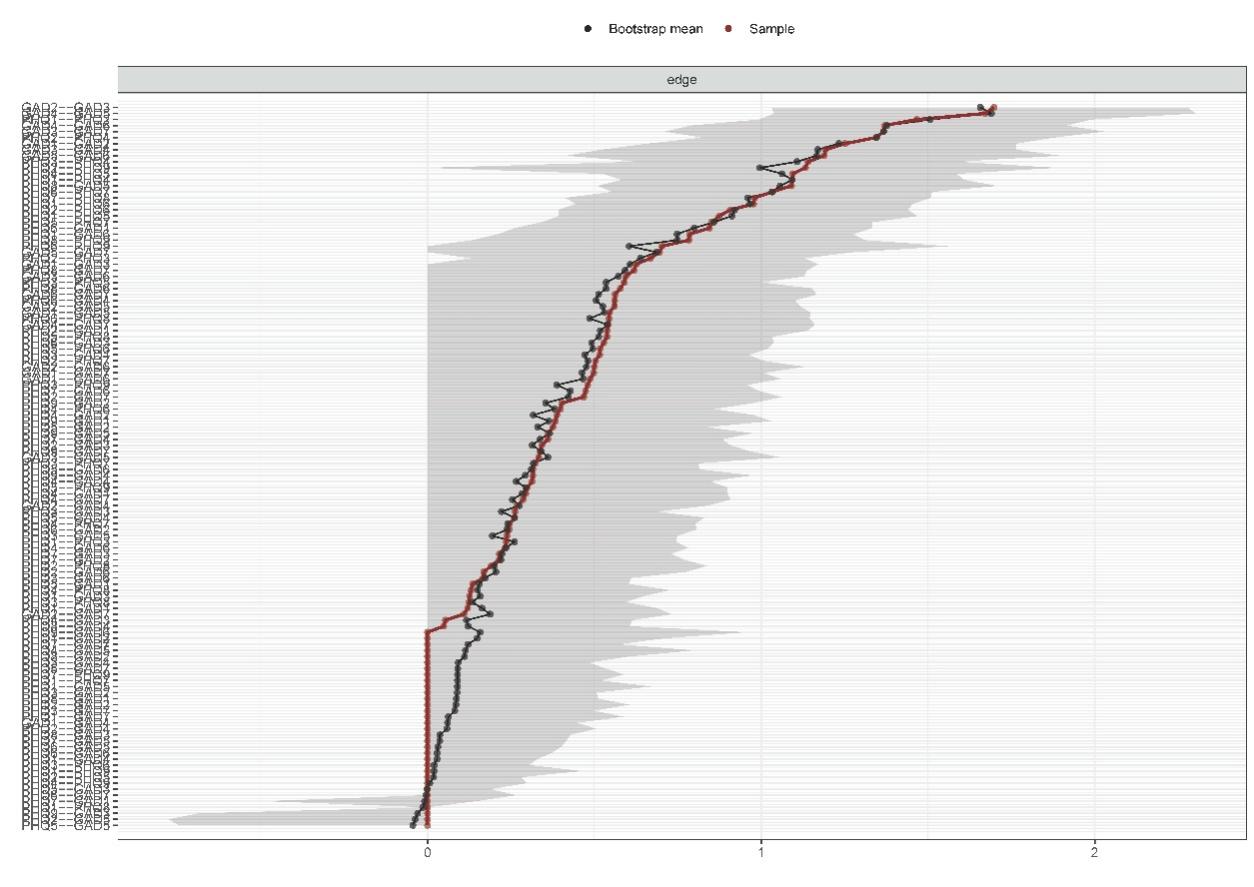


B:


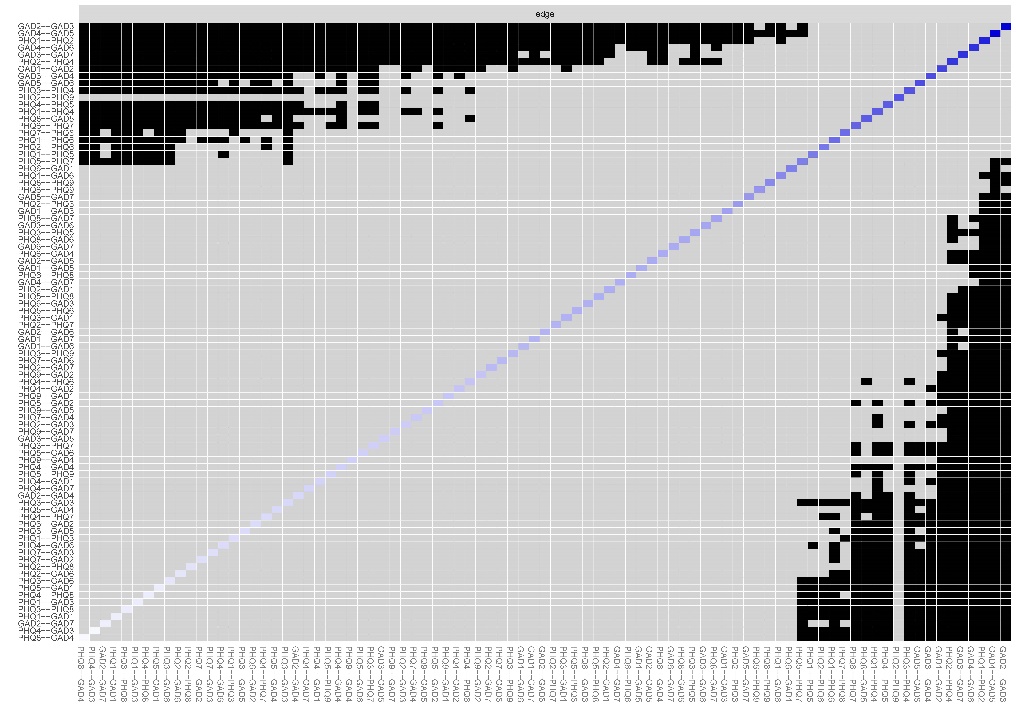


Figure S1. Panel A and Panel B: Nonparametric bootstrapped difference test for edge. For this, 95% bootstrapped confidence interval (CI) for edge-weights are constructed based on the normal variance in the bootstrapped sample. Based on the range of these CIs indicated that a wide interval represents low stability, and a network analysis interval represents high stability.

Figure S2. Estimated network model between males and females

A:


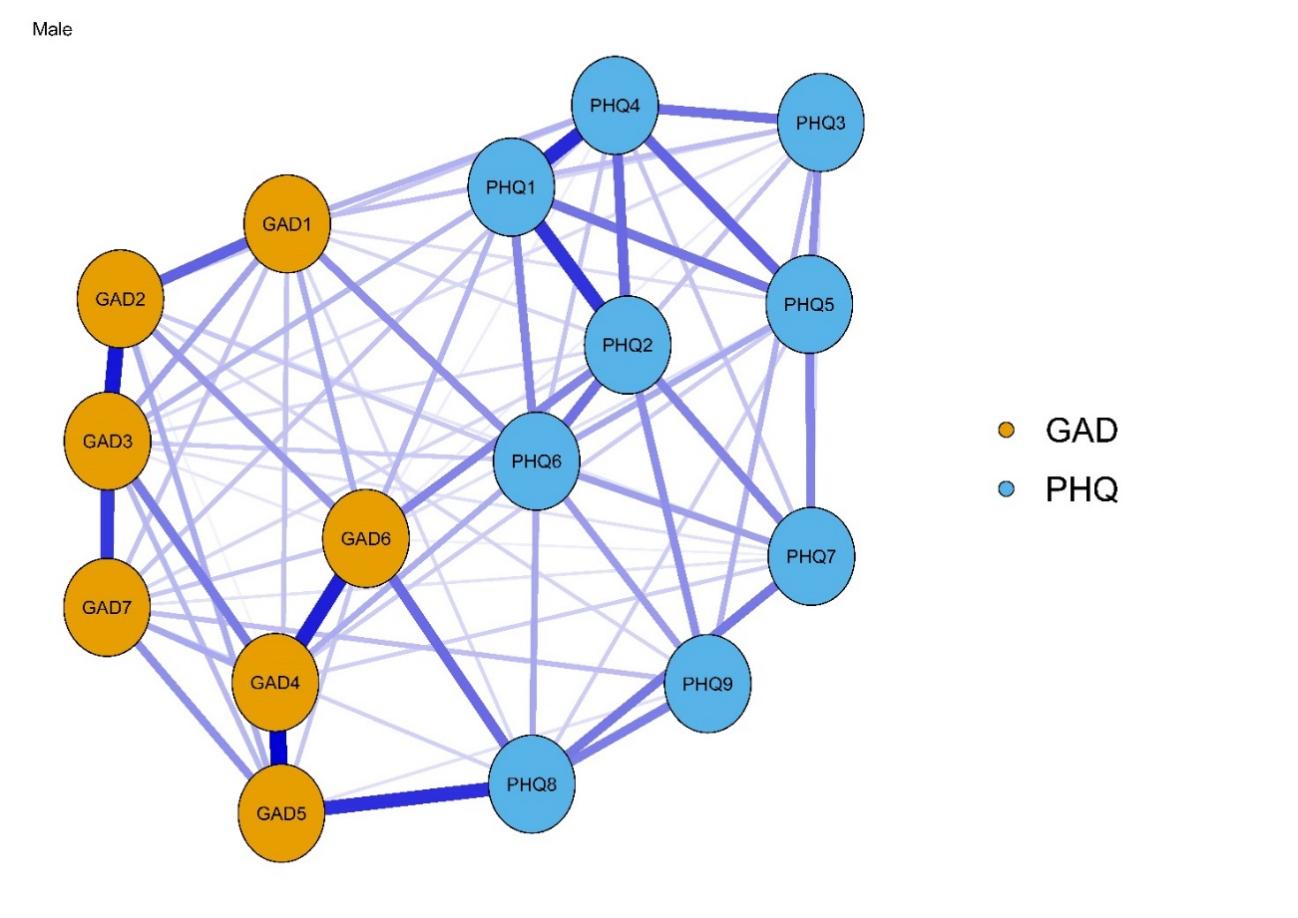


B:


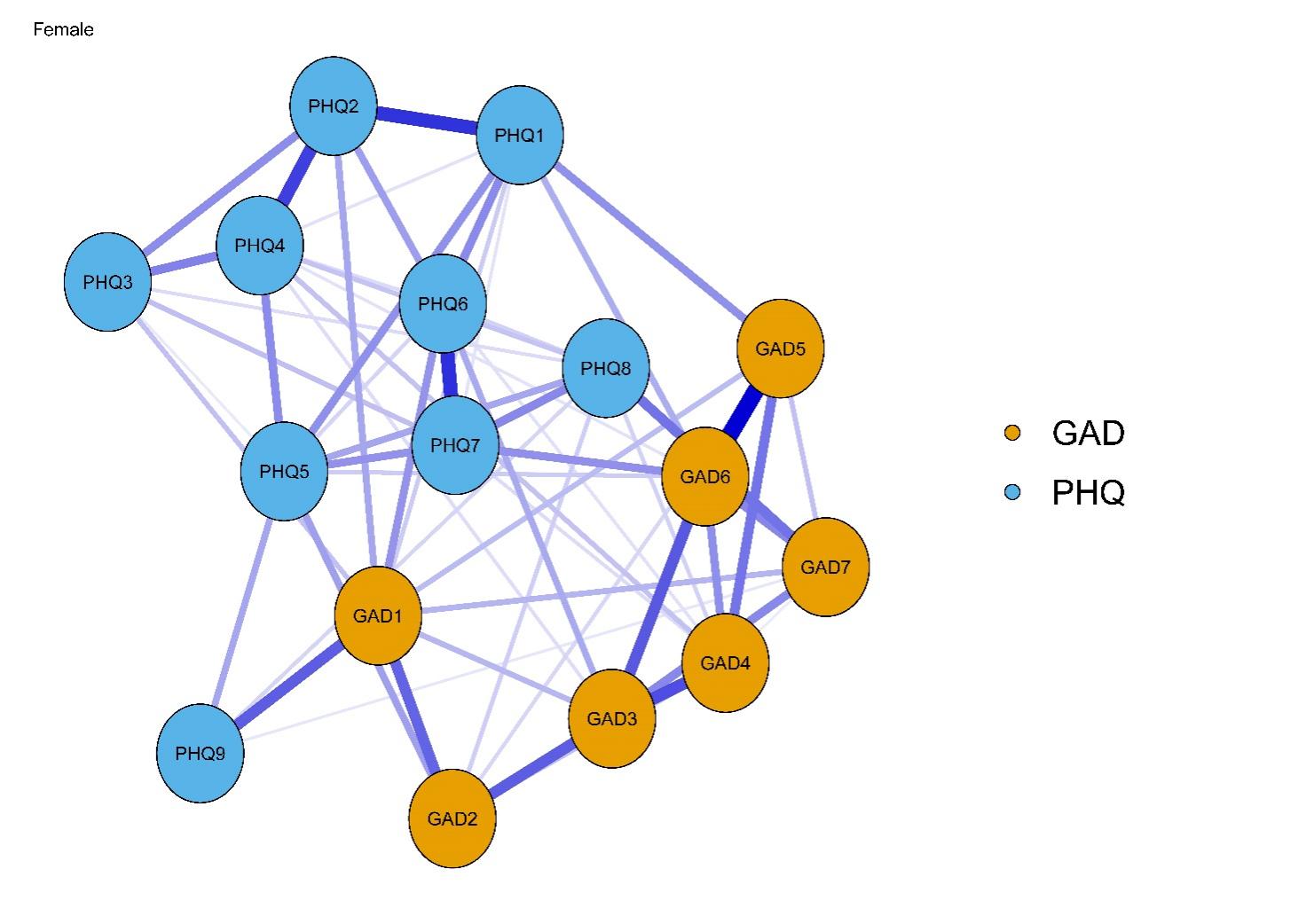


Figure S2. (A) Estimated network model for the association between depressive and anxiety symptoms in males (n = 420). (B) Estimated network model for the association between depressive and anxiety symptoms females (n = 637). The dark blue lines represent positive correlations. The edge thickness represents the strength of the association between symptom nodes.

Figure S3. Comparison of network centrality indices between females and males.


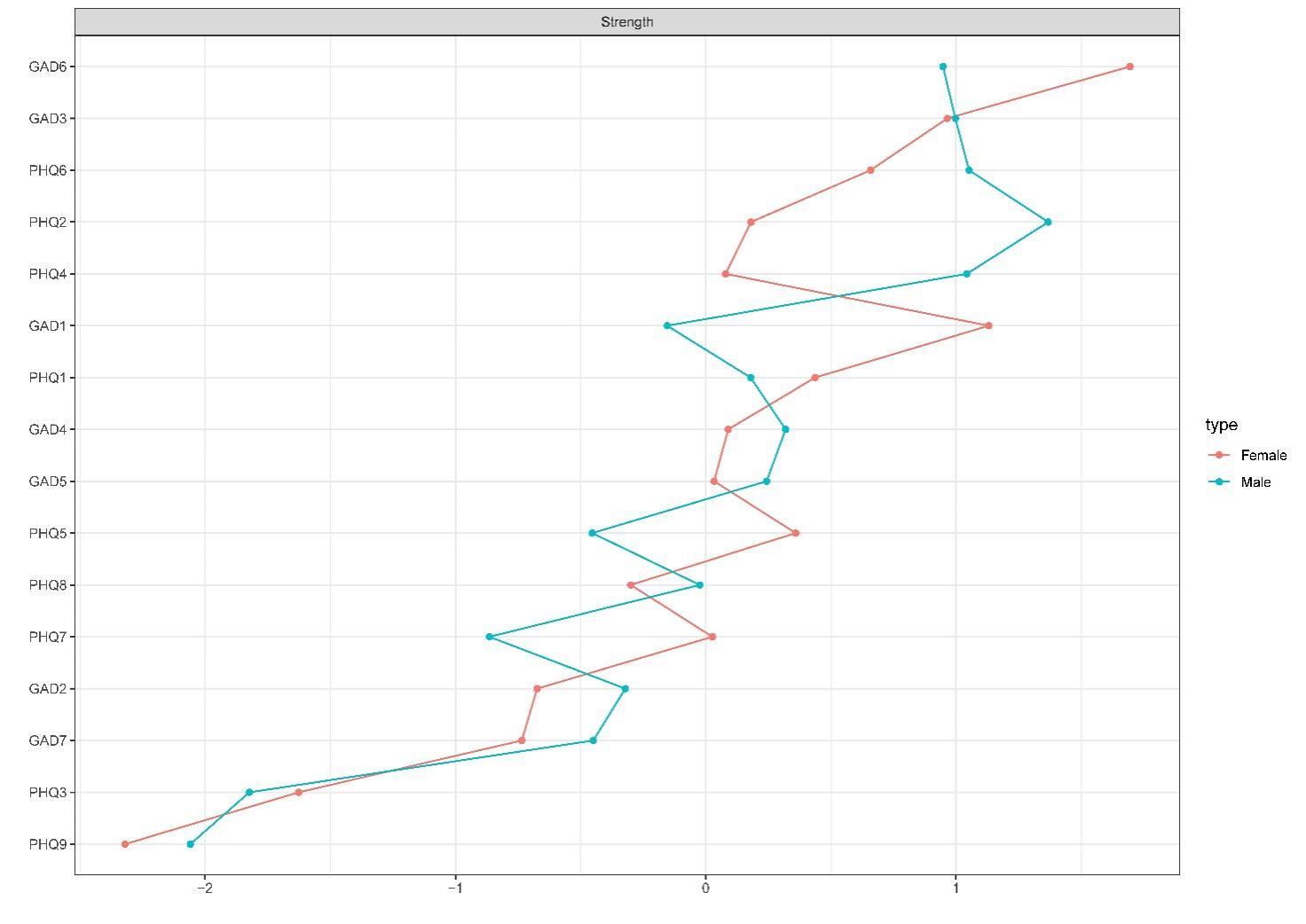


Figure S4. Comparison of network properties between females and males.

A:


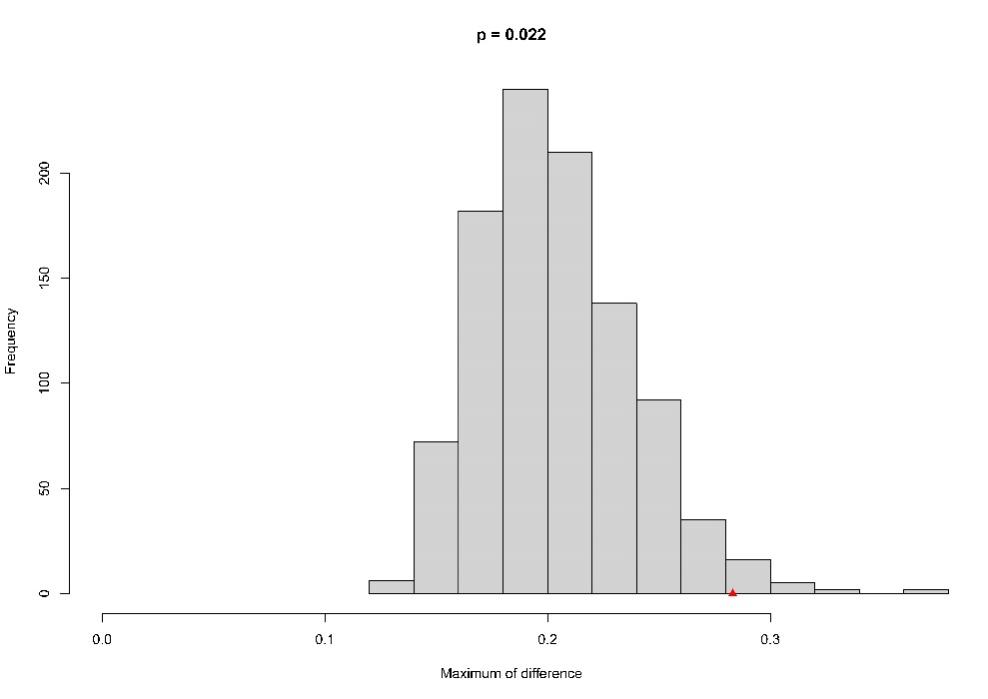


B:


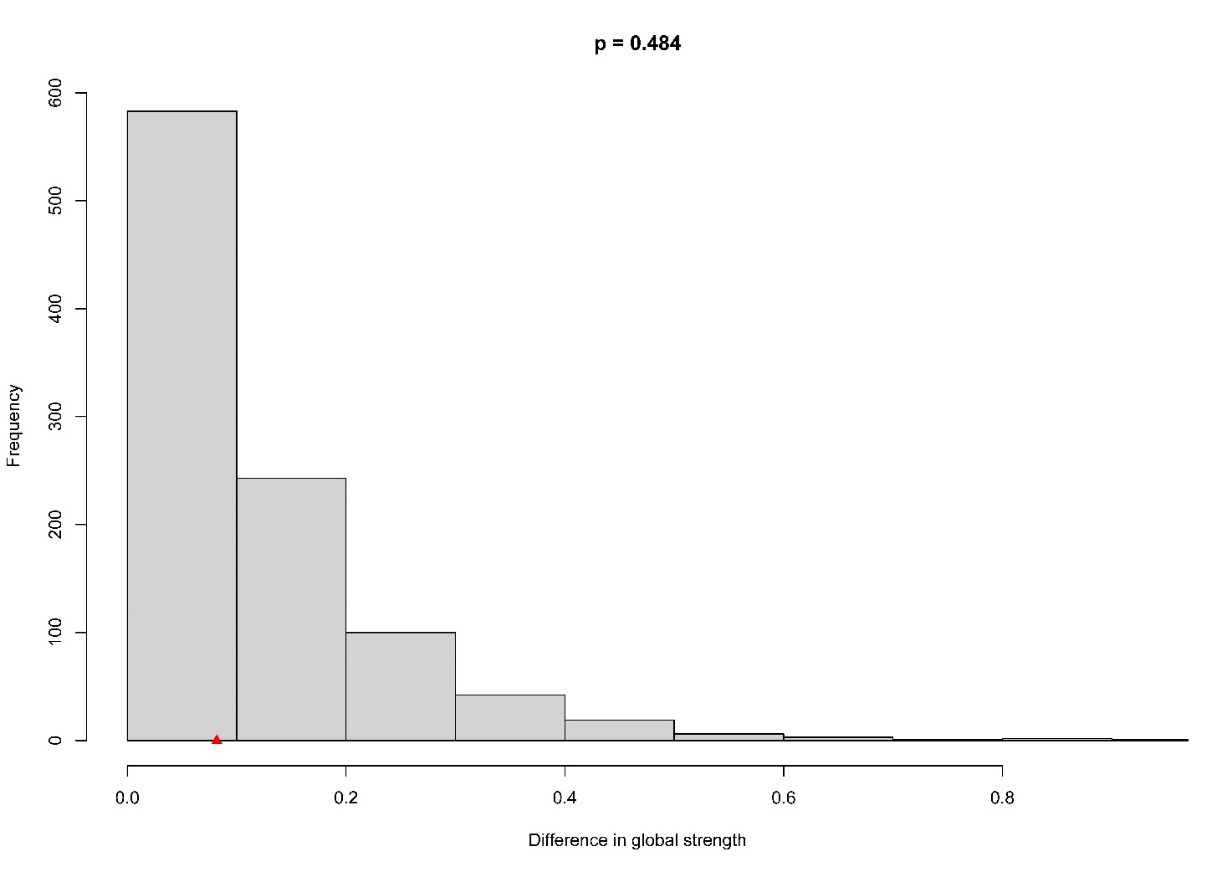


A Panel: A plot of bootstrap value of the maximum difference in any of the edge weights (1000 permutations). The difference was significant (M=0.28, p=0.022).

B Panel: A plot of bootstrap value of the difference in network global strength. The difference was not significant (network strength among male participants: 7.52; among female participants: 7.60; S: 0.08, p=0.484). Invariance in edge weights was tested using the permutation test, generating sets of p values for each edge-edge comparison. Holm-Bonferroni corrected p values were all >0.05 indicating absence of significant differences.

Figure S5. Estimated network model between junior and senior students.

A:


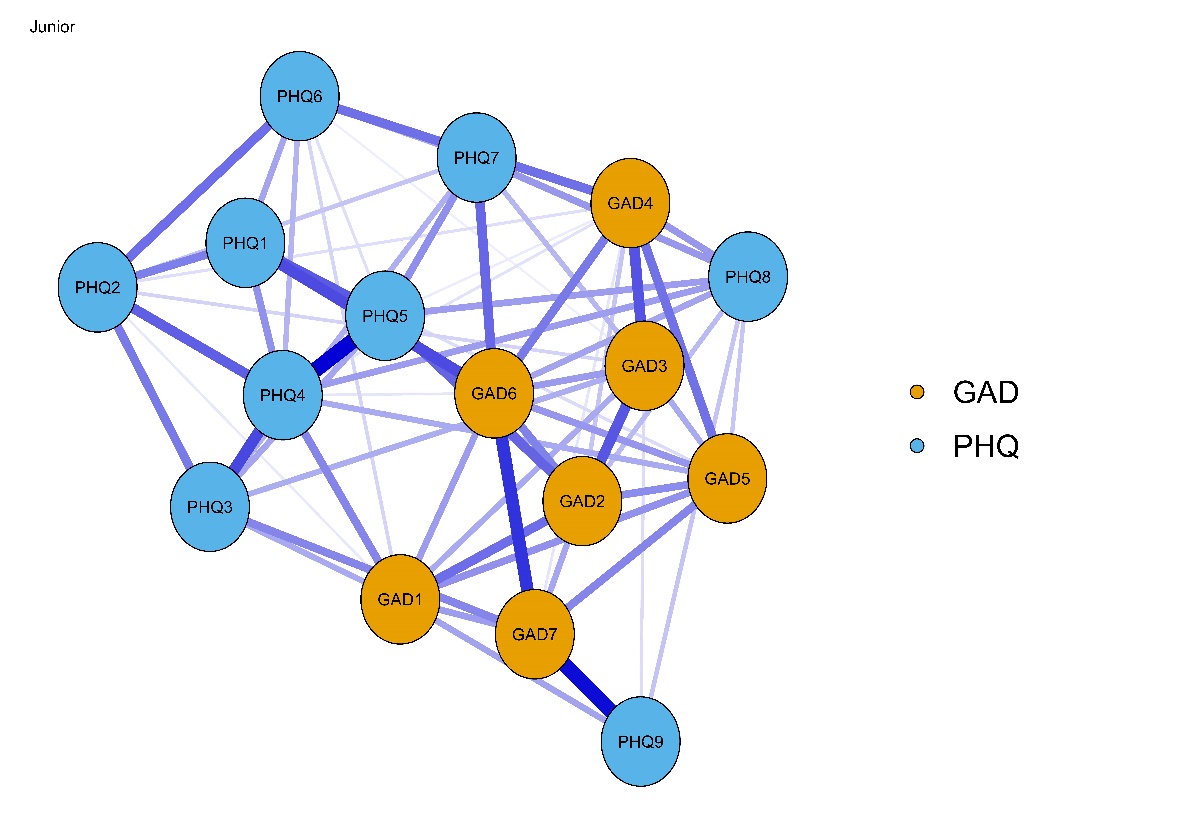


B:


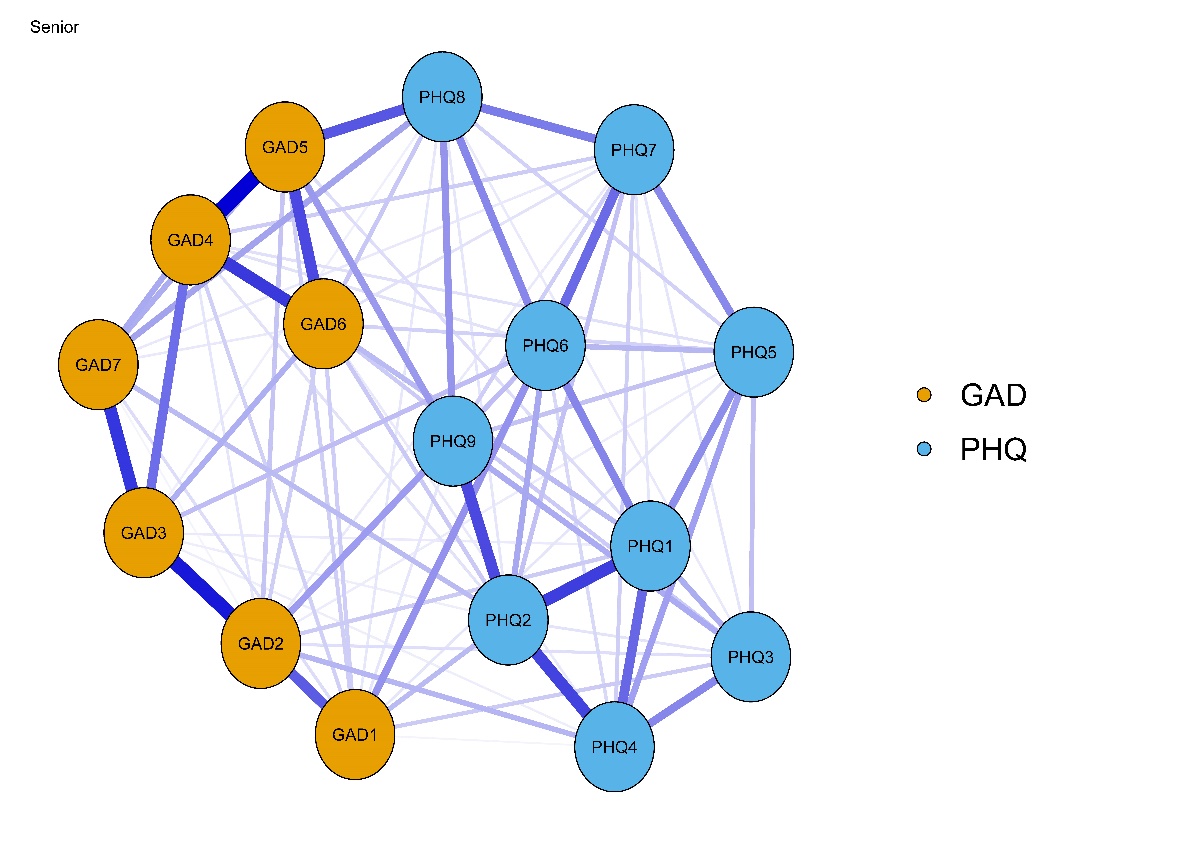


Figure S5. (A) Estimated network model for the association between depressive and anxiety symptoms in junior (n = 479). (B) Estimated network model for the association between depressive and anxiety symptoms in senior (n = 578). The dark blue lines represent positive correlations. The edge thickness represents the strength of the association between symptom nodes.

Figure S6. Comparison of network centrality indices between junior and senior students.


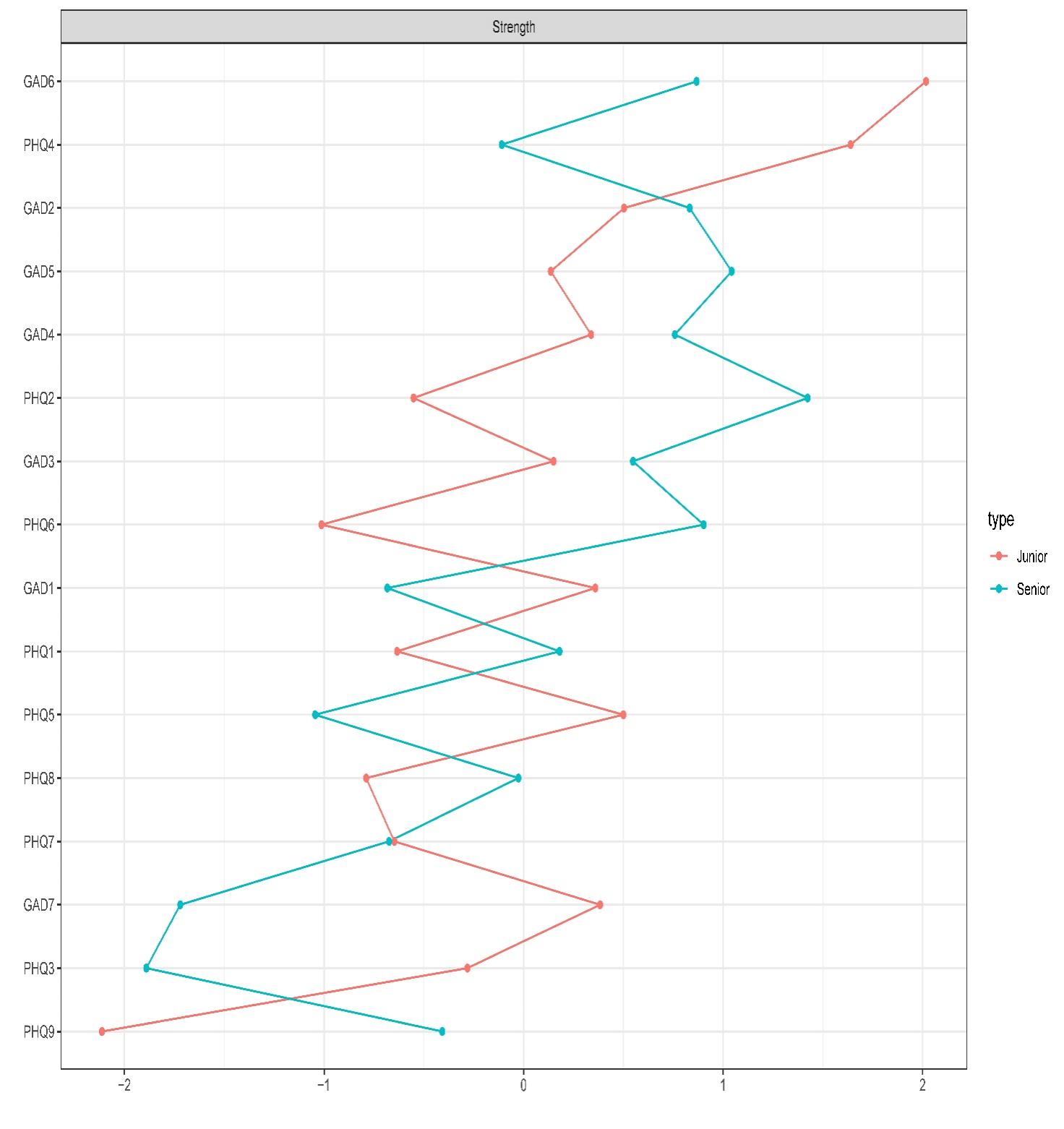


Figure S7. Comparison of network properties between junior and senior students.

A:


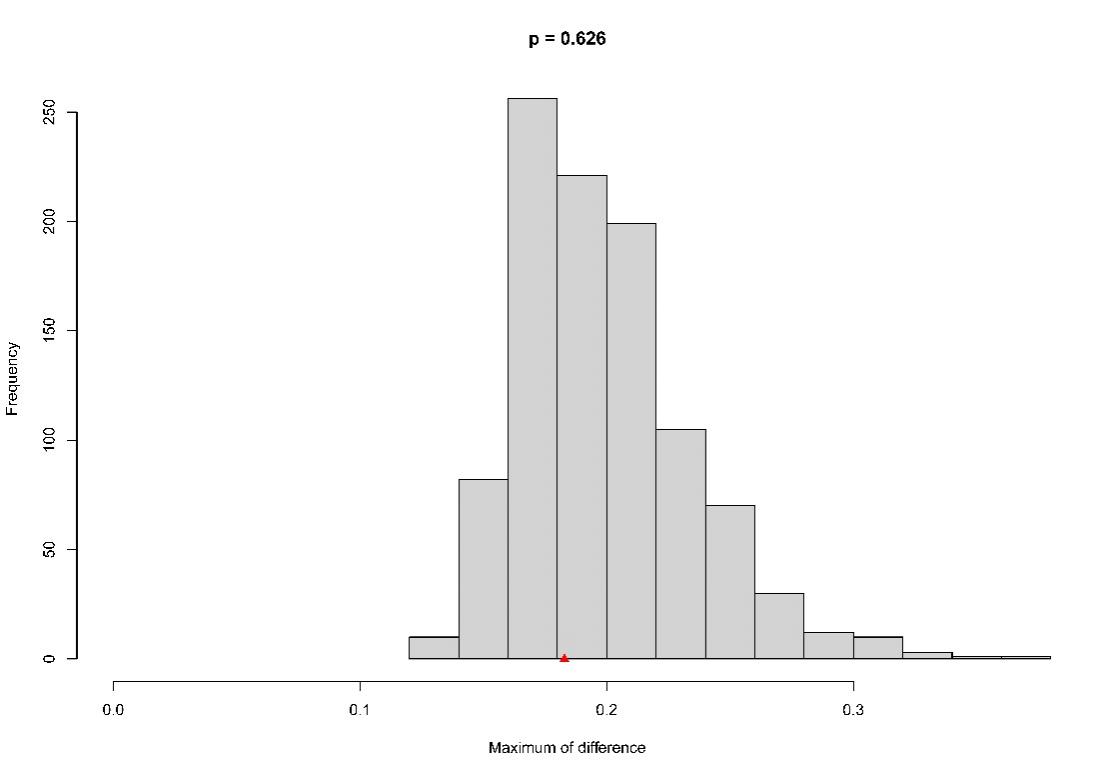


B:


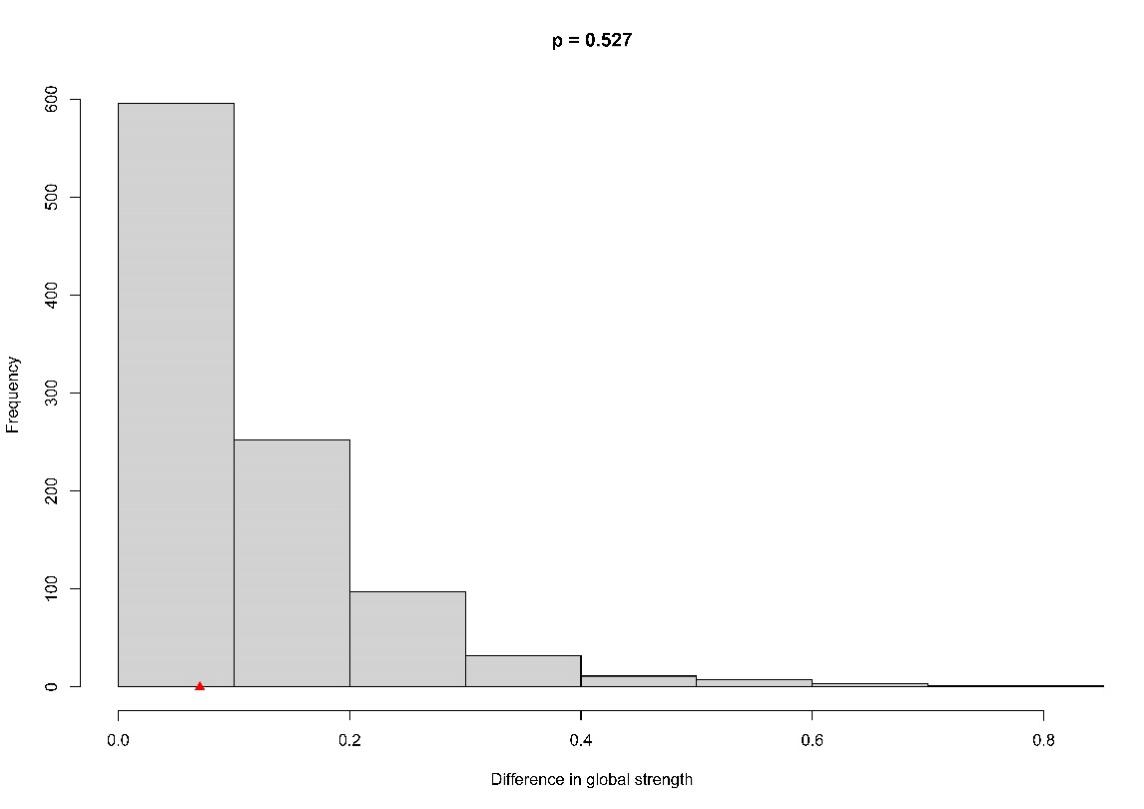


A Panel: A plot of bootstrap value of the maximum difference in any of the edge weights (1000 permutations). The difference was not significant (M=0.18, p=0.626).

B Panel: A plot of bootstrap value of the difference in network global strength. The difference was not significant (network strength among junior participants: 7.47; among senior participants: 7.40; S: 0.07, p=0.527). Invariance in edge weights was tested using the permutation test, generating sets of p values for each edge-edge comparison. Holm-Bonferroni corrected p values were all >0.05 indicating absence of significant differences.

Figure S8. Estimated network model between urban and rural students.

A:


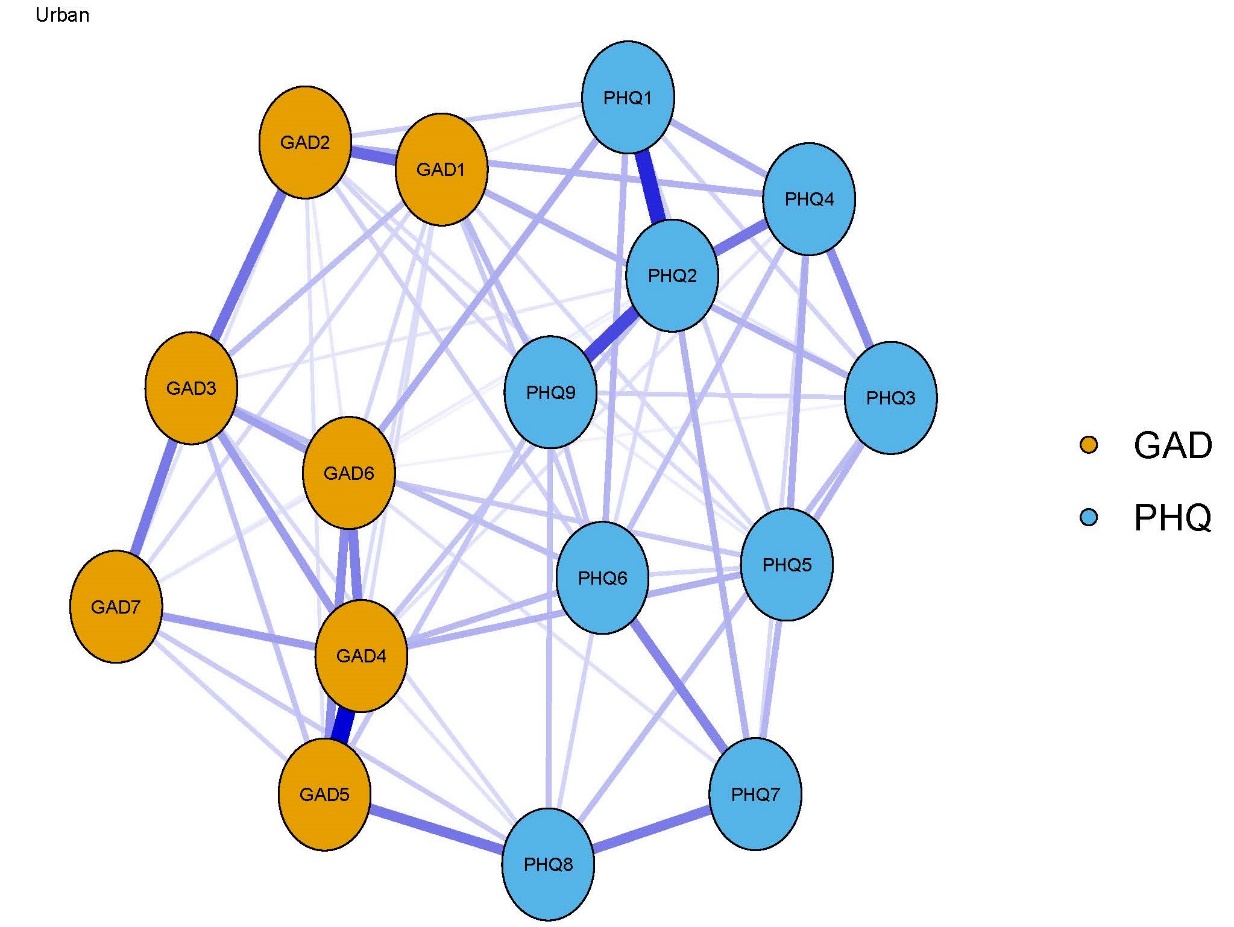


B:


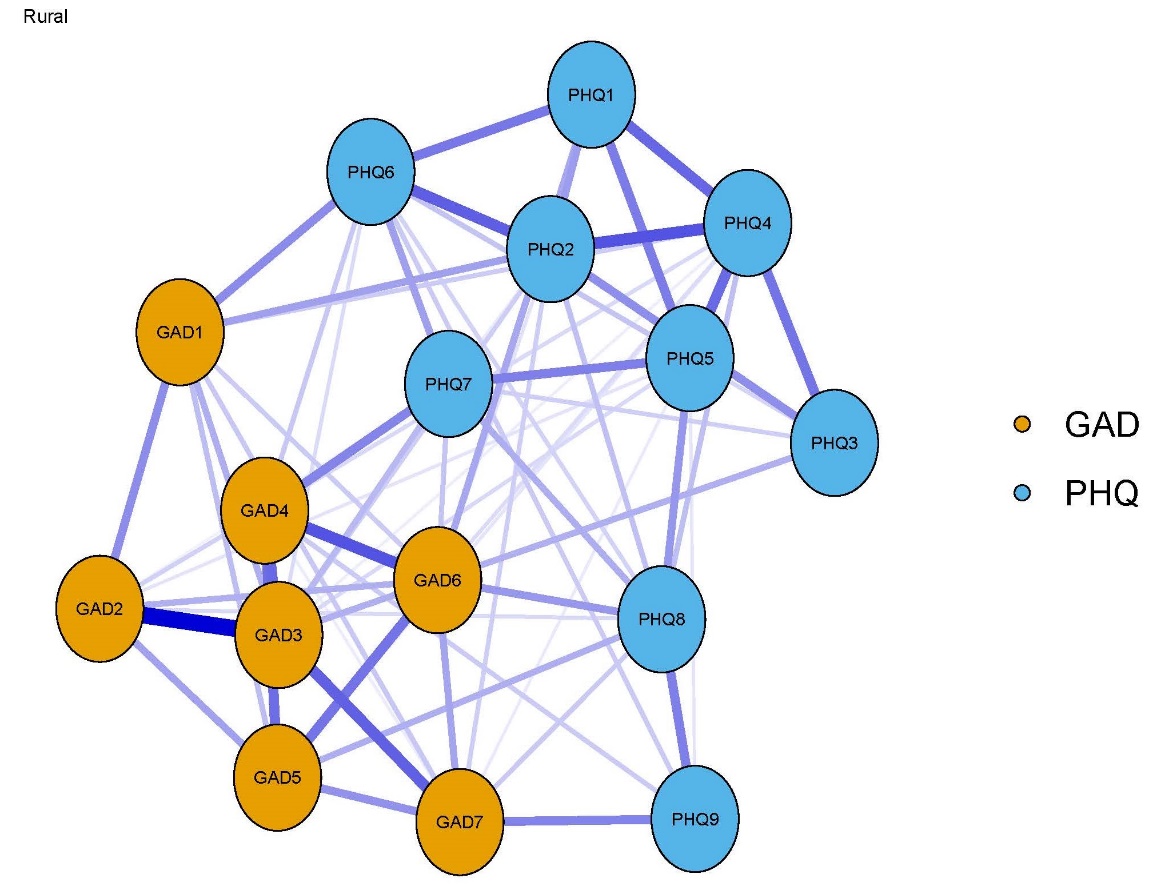


Figure S8. (A) Estimated network model for the association between depressive and anxiety symptoms in urban areas (n = 407). (B) Estimated network model for the association between depressive and anxiety symptoms in rural areas (n = 650). The blue nodes denote the PHQ items; the yellow nodes denote the GAD items. The dark blue lines represent positive correlations. The edge thickness represents the strength of the association between symptom nodes.

Figure S9. Comparison of network centrality indices between urban and rural students.


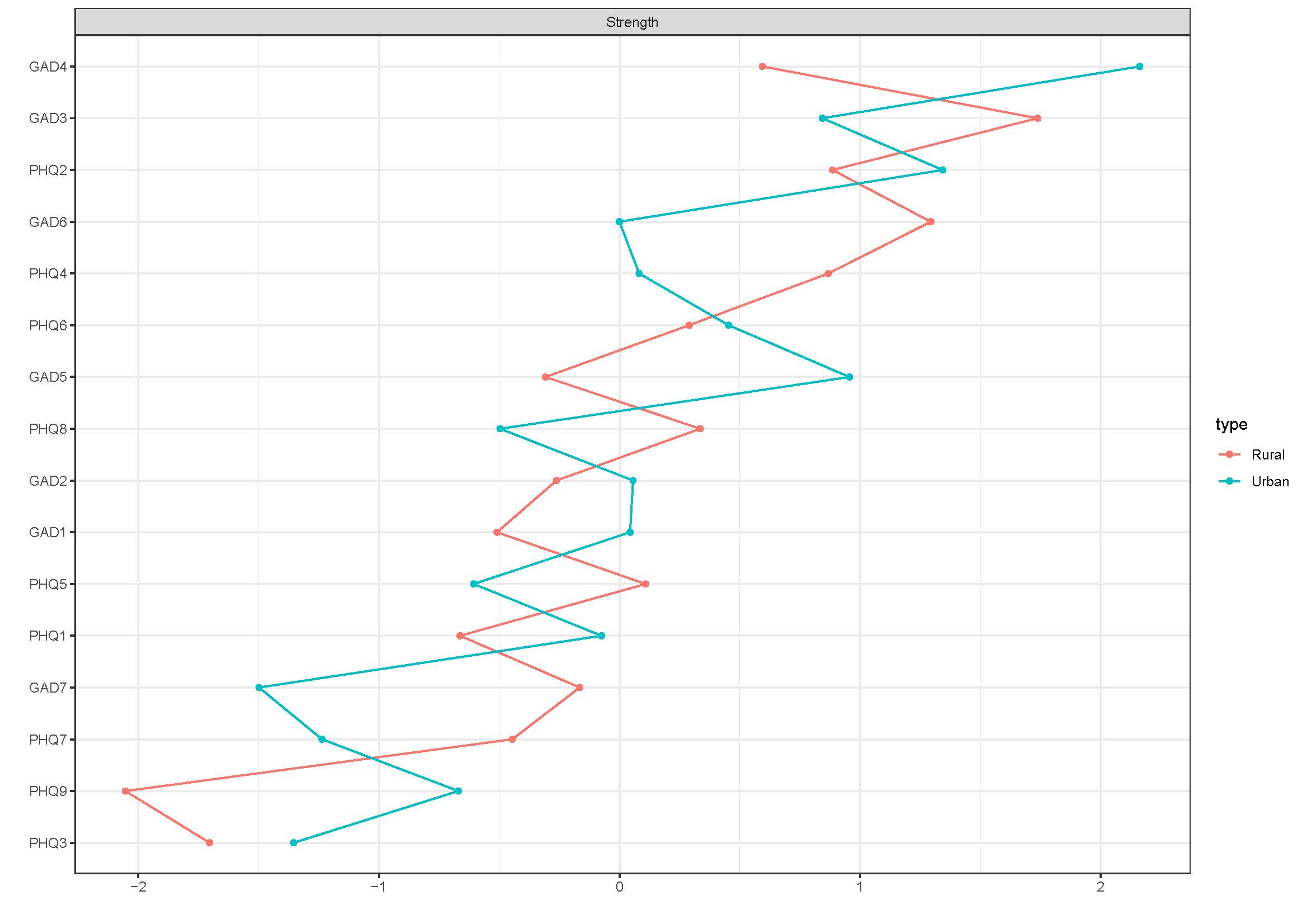


Figure S10. Comparison of network properties between urban and rural students.

A:


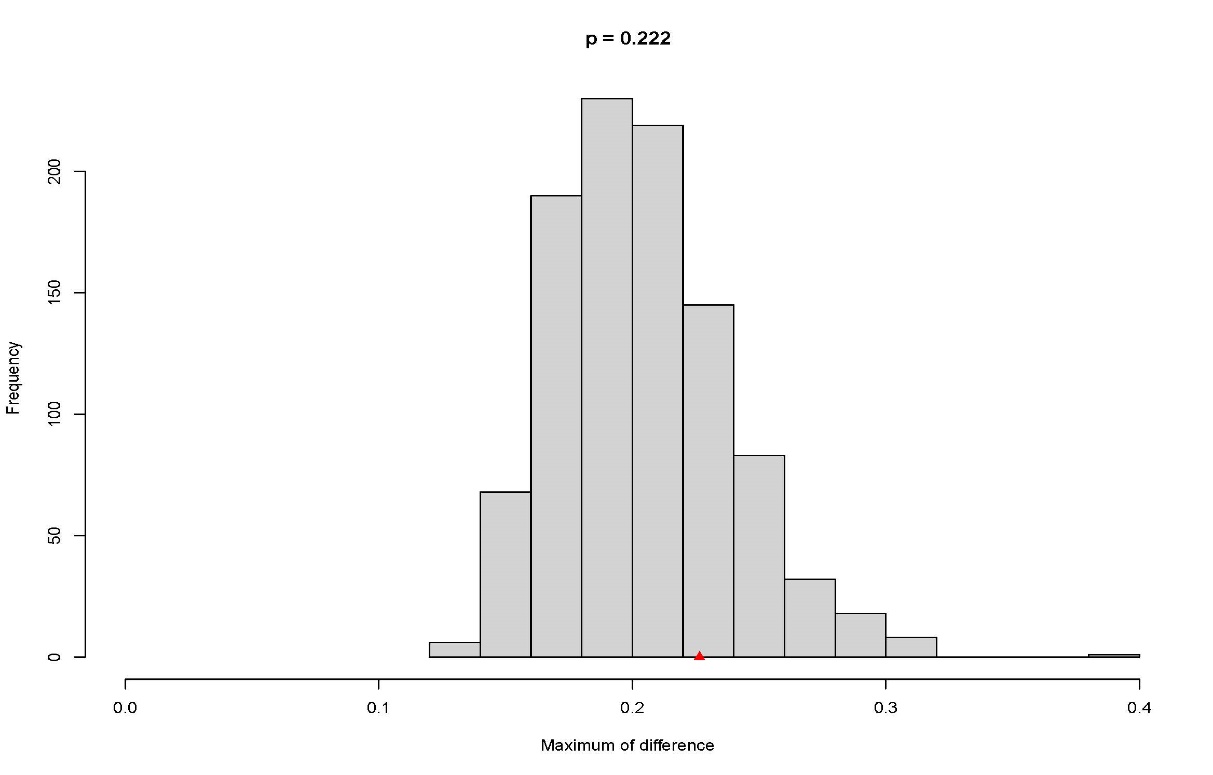


B:


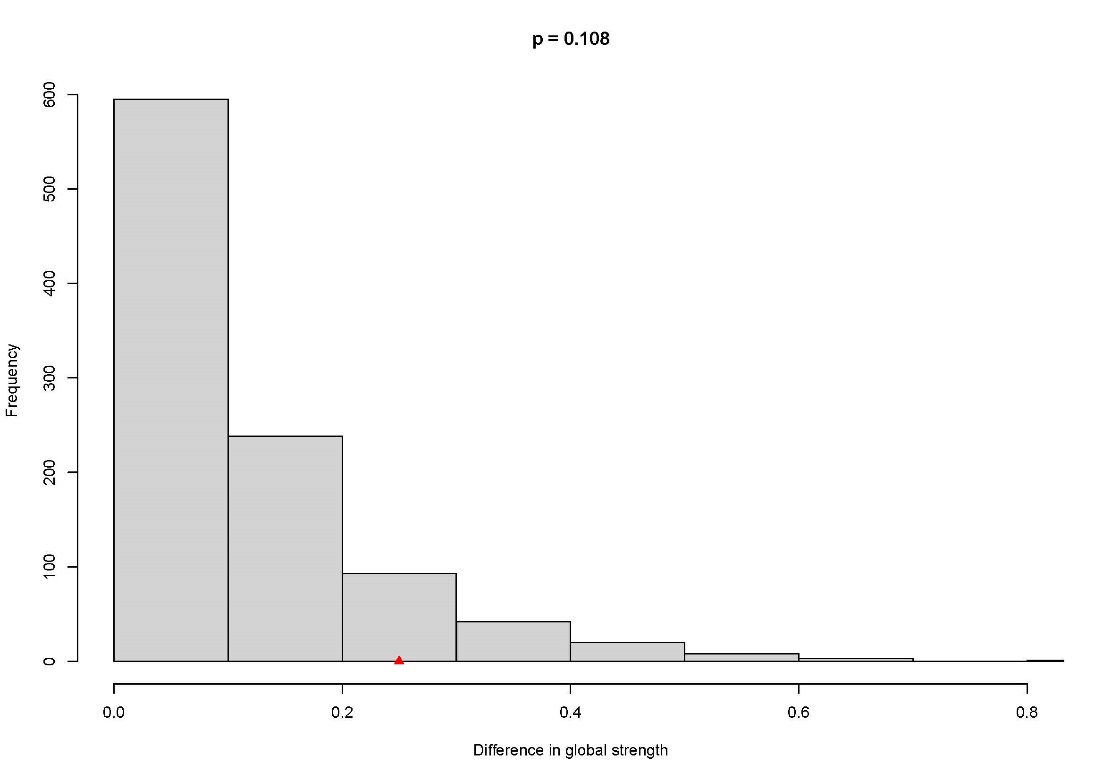


A Panel: A plot of bootstrap value of the maximum difference in any of the edge weights (1000 permutations). The difference was not significant (M=0.22, p=0.222).

B Panel: A plot of bootstrap value of the difference in network global strength. The difference was not significant (network strength among urban participants: 7.36; among rural participants: 7.61; S: 0.25, p=0.108). Invariance in edge weights was tested using the permutation test, generating sets of p values for each edge-edge comparison. Holm-Bonferroni corrected p values were all >0.05 indicating absence of significant differences.
